# Comparison of CT-measured angles of pelvic limbs without patellar luxation of six canine breeds

**DOI:** 10.3389/fvets.2023.1194167

**Published:** 2023-07-13

**Authors:** Andreas Brühschwein, Juliette Burg-Personnaz, Martin Zöllner, Sven Reese, Andrea Meyer-Lindenberg

**Affiliations:** ^1^Clinic of Small Animal Surgery and Reproduction, Centre of Veterinary Clinical Medicine, Veterinary Faculty, LMU Munich, Munich, Germany; ^2^Institute of Veterinary Anatomy, Histology and Embryology, Department of Veterinary Sciences, Veterinary Faculty, LMU Munich, Munich, Germany

**Keywords:** dog, computed tomography, hind limb, angulation, measurement, breed-related, torsion, valgus

## Abstract

**Introduction:**

Dogs with medial patellar luxation can be affected by pelvic limb deformities whose corrective osteotomies and associated biomechanical rebalancing might provide higher success rates than standard surgical procedures limited to the stifle joint. In bilaterally affected canine patients, comparison with the contralateral normal limb is impossible. Reference values are useful for orthopedic decision-making. Inconsistency of published reference values might depend on methodology or canine breed. We hypothesized that canine pelvic limb alignment is breed-specific.

**Methods:**

CT scans of 42 pelvic limbs of dog breeds predisposed for medial patellar luxation, with an orthotopic patellar position and stability were studied. Several angleswere measured with an open-source 3D Slicer plugin using vector calculations. The breeds were compared with a general linear model with a Bonferonni adjustment using SPSS.

**Results:**

Chihuahuas, Pomeranians, Jack Russel Terriers, Pugs, French Bulldogs, Maltese were examined. In the order of the listed breeds, the angles were as follows: 28.3°±10.7°, 20.1°±2.9°, 35.4°±6.9°, 32.8°±3.0°, 19.0°±7.1°, 26.6°±5.3° for the antetorsion, 5.3°±1.8°, 2.8°±2.8°, 8°±4.4°, 3.8 °±3.1°, 4.7°±3.3°, 2.3°±3.3° for the femoral varus, of −5.5°±6.2°, 1.1°±4.1°, −5.2°±9.5°, 6.1°±8.0°, −0.1°±5.9°, −9.2°±4.7° for the tibial torsion, 2.0°±2.9°, 2.1°±2.7°, 6.4°±6.8°, 0.0°±5.7°, 3.0°±5.8°, 8.8°±8.6° for the tibial valgus, 1.2°±10.4°, 1.8°±3.4°, −1.7°±4.9°, −1.7°±9.4°, 5.1°±8.8°, −0.2°±8.6° for the femorotibial rotation and −3.4°±2.2°, 1.1°±4.1°, −2.8°±3.4°, −5.2°±4.0°, −2.1°±4.4°, −5.4°±3.7° for the tibiotalar rotation. There were significant differences between breeds in femoral torsion, femoral varus, and tibial torsion angles, but no significant differences in tibial valgus, femorotibial, and tibiotalar rotation angles.

**Discussion:**

Our hypothesis is therefore partially correct. Our results are limited to small dogs prone to medial patellar luxation and might not be generalized. To establish robust reference values larger case numbers and more breeds should be evaluated. In conclusion, canine pelvic limb alignment reference values for small dogs with a predisposition for medial patellar luxation should be considered breed-specific.

## Introduction

Patellar luxation in dogs is a common orthopedic disorder in small animal practice and is considered to be of congenital, developmental, or traumatic origin (1, 2). Patellar luxation can occur unilaterally or bilaterally, with intermittent medial, lateral, or bidirectional occurrence, and is graded in 4 severity levels (1, 2). Medial patellar luxation occurs more frequently than lateral luxation and is more common in small dogs than in larger dogs, where lateral patellar luxation is more common (1, 2). Although environmental factors may play a role, medial patellar luxation may be a polygenic disorder with a heritable etiology, supported by a high proportion of bilateral nontraumatic cases and breed predisposition (1, 2). Pomeranian, Pug, Chihuahua, Maltese, Poodle, Shih Tzu, West Highland White Terrier, Yorkshire Terrier, Jack Russell Terrier, Bull Terrier, French Bulldog, Cavalier King Charles Spaniel and other breeds are predisposed for medial patellar luxation (3). Flat Coated Retriever, Labrador Retriever, Husky, or Boxer are examples for large breed dogs with dispositions for lateral patellar luxation (1, 2). In dogs with patellar luxation, soft tissue aspects play an important role in the pathophysiology, and common surgical treatment techniques are partially-based on rebalancing soft tissue procedures of the stifle joint. These surgeries include retinacular incision and desmotomy on the side of patellar luxation, where soft tissue tightening occurs, and retinacular overlap and imbrication on the stretched and weakened contralateral side of the joint capsule and retinaculum (1, 2). Other commonly used surgical treatment techniques are bony procedures, such as tibial tuberosity transposition surgery, which corrects malposition of the tibial tuberosity causing malalignment of the muscular quadriceps mechanism (1, 2). Poor medial alignment of the tibial tuberosity can be caused by femoral-tibial stifle joint rotation, bony misalignment of the tibial tuberosity relative to the diaphysis, and tibial torsion deformity (4). Other common osseous procedures are the various trochleoplasty techniques, which aim to correct a flattened trochlear groove by deepening and modifying the shape of the trochlear groove (1, 2). Most cases of medial patellar luxation are considered to be of developmental origin. Besides pathologic soft tissue alterations impacting angular limb alignment, osseous deformities commonly occur in dogs with patellar luxation and might play an important role in pathophysiology, representing a complex skeletal malformation, rather than an isolated stifle joint disease (1, 2, 4–19). To characterize canine hindlimb alignment and to understand, assess, and quantify the severity of patellar luxation, multiple angular measurements have been performed using diagnostic imaging. Alterations in femoral neck version as well as abnormal femoral and tibial torsion and femoral varus angles may be associated with patellar luxation (1, 2, 5–13, 15–17, 19–25). Angular measurements are often performed using planar two-dimensional radiographs to determine bone deformities in the canine femur (9, 13, 16–18, 21, 26, 27) and tibia (28–35). But the radiographic limitations including superimposition, magnification, and distortion decrease the quality of assessment of bone deformities (36). As demonstrated by the distal femoral varus angle, small changes in positioning can affect angular measurements (37–41).

Angular measurements of femoral and tibial torsion angles using two-dimensional radiography are technically difficult (20–22). Computed tomography (CT) generates true three-dimensional (3D) data and can be used to overcome the limitations of radiography (2, 9, 11, 12, 14, 18, 26–44). A coplanarity of reference axes is required to measure an angle, but anatomical reference points can be located in several primary transversal images. Therefore, different techniques are used to enable measurements within more than one two-dimensional image. Post-processing techniques such as multiplanar reconstruction (MPR) (12, 45–47), maximum intensity projections (MIP) (45) and volume rendering technique (VR) (1, 17, 38, 48–55) can be used to enable angular measurements in CT scans. A variable alignment of the image plane is possible using a MPR, but the image and angular measurement remains two-dimensional (12, 45–47). Using MIP (45) and VR (1, 17, 38, 48–56), free manual object rotation and an adjustable choice of perspective are possible. This equals a virtual radiographic positioning, so the final image is still two-dimensional, resulting in a coplanar projection plane selected by the operator based on his visual and subjective orientations. In addition to radiography and CT, MRI can be used in a clinical setting to measure canine pelvic limb angles (19, 23). Besides different imaging modalities, several anatomical angles, based on various axes and anatomical reference points are described to determine canine femoral and tibial alignment angles (57).

According to a systematic review several studies agree, that in the frontal (dorsal) plane the degree of malalignment of the distal femur and as well as proximal and distal tibia correlate with the severity of medial patellar luxation (1, 45). Especially for patients with bilateral bone deformation, the importance of reference values for individual breeds was pointed out (1). Reference values for several canine breeds are reported and reviewed, but are based on various technical approaches for angular measurements including different imaging modalities and their inherent technical implications (45). In human medicine, the reference value of femoral torsion angle, even within the same modality, refers only to the corresponding measurement technique used and cannot be transferred between different measurement techniques (58, 59). The authors of this study also noted the limitations of 2D measurements and questioned their accuracy (41). Dogs with complex angular hind limb deformities need precise morphological evaluation using diagnostic imaging to result in successful orthopedic surgery (28, 39, 60). Dogs affected by high-grade patellar luxation with severe bone deformities might benefit from additional corrective osteotomies (3, 16, 61), and in case of bilateral deformity reference values become important. Reference values might depend on measurement methodology and on the breed.

In this study, we aimed to support the hypothesis that canine pelvic limb alignment is breed-specific.

## Materials and methods

The clinical database of the hospital information system and the picture archiving and communication system of the Clinic of Small Animal Surgery and Reproduction, Center of Veterinary Clinical Medicine, LMU Munich and the private clinic of small animal Zebrasoma, Strasbourg were queried and documents of orthopedic examinations and computed tomographic studies were searched from 2008 to 2022. Inclusion criteria were pelvic limb CT-scans in caudally extended hind limb position of small dogs from breeds that are predisposed for medial patellar luxation with normal orthopedic examination of the stifles with a stable orthotopic patella at the same time and without prior orthopedic surgical procedure at the respective limb. CT-data should contain thin-sliced, gap-free, high-resolution bone scans from the coxofemoral joints to the metatarsal bones. Based on these inclusion criteria, we selected 42 limbs from 6 different breeds: 10 limbs of French Bulldogs, 8 of Chihuahuas, 6 of Pugs, 5 of Maltese, 6 of Pomeranians and 7 of Jack Russell Terriers. CT scans were performed using a helical multi-slice CT scanner (Somatom Definition AS VA48A_02_P12, 64 Excel Ed. software Somaris/7 syngo CT VA48A Siemens Healthcare GmbH, Erlangen, Germany and Canon Medical Aquilion Lightning, Software version V10.20FR005, Canon Medical Systems Europe B.V., Zoetermeer, Netherlands) in a helical mode. Detector slice thickness was set at 0.6 mm, tube voltage at 120 kV, tube rotation time at 0.5 – 1 s, pitch at 0.6 – 1 with tube currents variably adjusted according to the size of the patient. The reconstructed slice thicknesses and increments were the same, ranging between 0.6 mm and 0.75 mm, creating gap-free image stacks and therefore, continuous true 3D CT data. Images were reconstructed using a bone algorithm (deconvolution filter: kernel 60 or 70). Patients were in dorsal or ventral recumbency with extended pelvic limb, similar to a pelvic radiograph for canine hip dysplasia screening (OFA-view), but we did not require a perfect symmetry or a full extension of the pelvic limb. DICOM-images were imported into 3D Slicer software (Version 4.11.20210226, www.slicer.org) (49, 56).

Anatomical reference points for angle measurement were set in the CT data, using three orthogonal planes and 3D volume rendering CT images in a standard bone window. Coordinates of the reference points in a 3D Cartesian coordinate system were used to calculate the anatomical angles based on vector calculations, and vectors of anatomical axes were projected into mathematically defined projection planes. Measurements of femoral torsion, femoral varus (or valgus), femorotibial rotation, tibial torsion, tibial varus (or valgus), and tibiotalar rotation angles are vector-based calculations using an open-source 3D Slicer plugin written in the Python programming language (46). Our self-written open-source 3D Slicer plug-in software has been validated in previous studies using commercial 3D medical imaging software to measure canine hindlimb alignment (62–64).

Angle calculations necessitate vectors and planes which were defined by manually set points. Six angles were calculated for each limb. From proximal to distal: the antetorsion angle, the varus or valgus angle of the femur, the femorotibial rotation angle, the tibial torsion, the varus or valgus angle of the tibia, and the tibiotalar rotation angle were measured.

**Figure 1:**
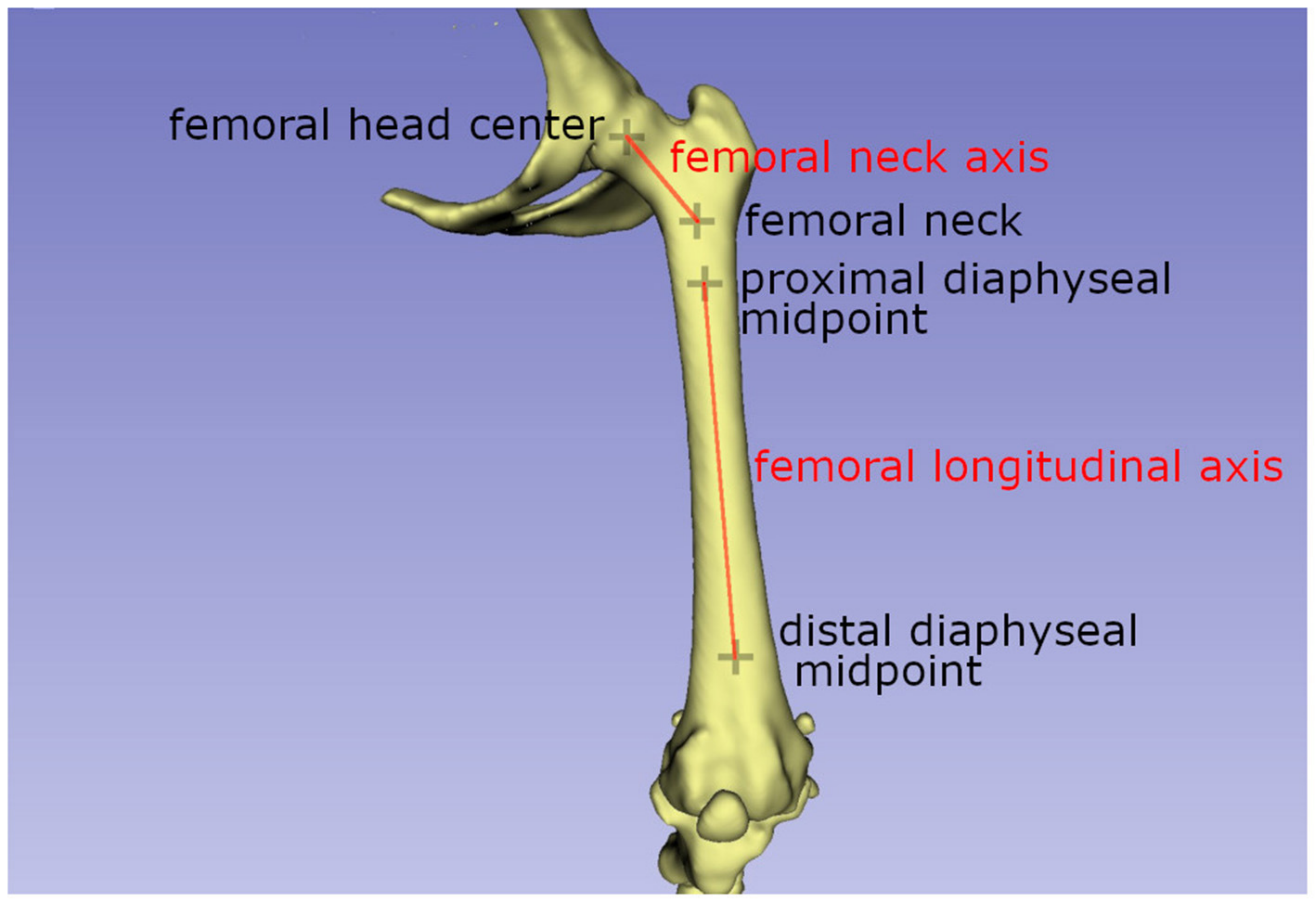
Schematic drawing explaining the axis used to calculate the femoral antetorsion angle.The femoral neck axis is the line between the femoral head center, calculated by sphere fitting (projected from another plane) and the femoral neck center. The femoral longitudinal axis is the line connecting the proximal and distal diaphyseal midpoints.

The femoral antetorsion angle:

The antetorsion angle was calculated as described previously by Barnes et al. (27). The center of the femur head was calculated by fitting a sphere using five points set along the capital bearing area (47). The center of the proximal femoral metaphysis on the height of the highest elevation of the lesser trochanter was selected as the femur neck basis center (Figure 1). The lateral and medial condyle points were set manually at the midpoint of the most caudal and distal point on the convex condyle surface (Figure 1). Both vectors were projected in the transversal femoral plane where the angle was calculated (55).

**Figure 2:**
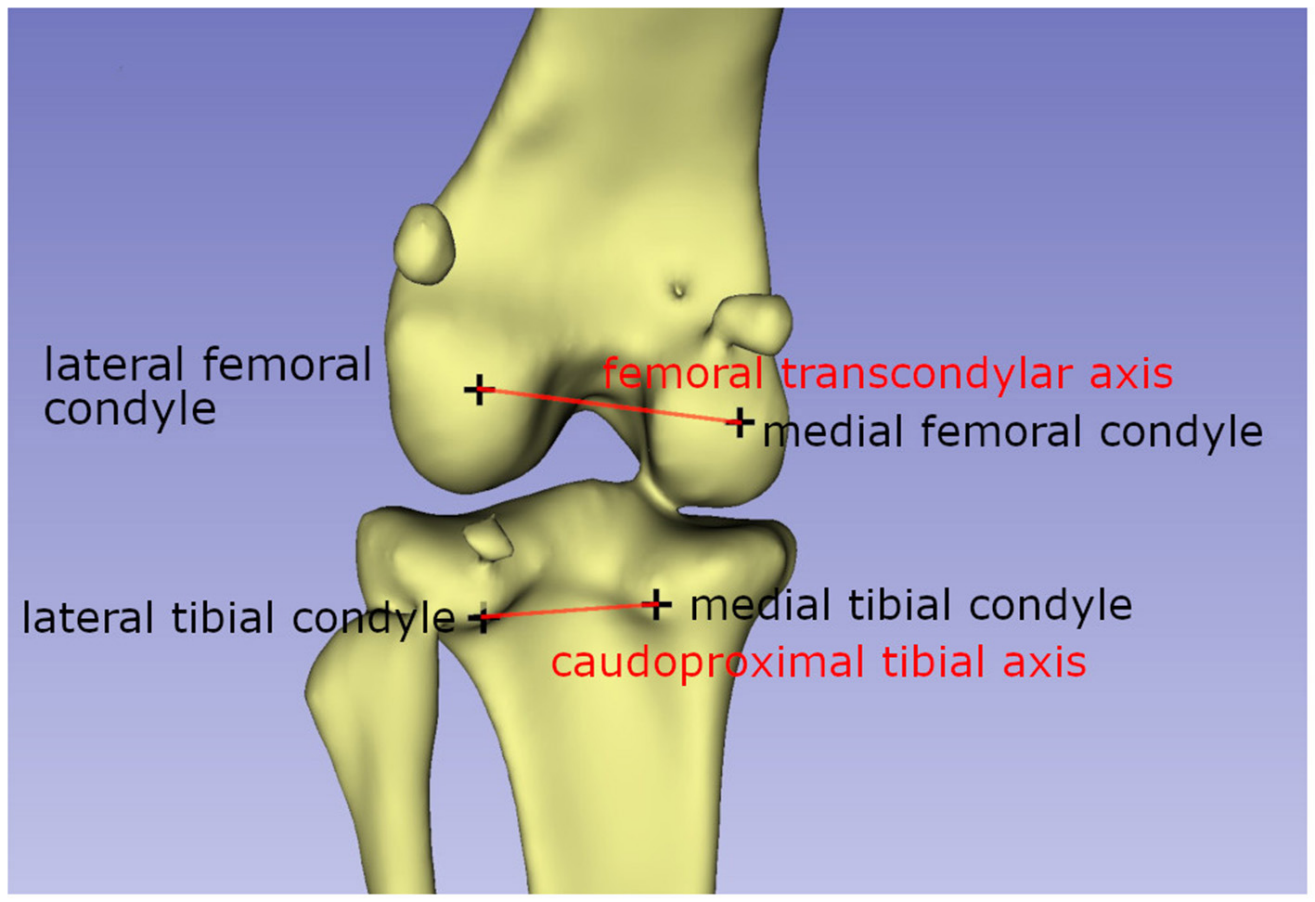
Schematic drawing explaining the axis used to calculate the femorotibial rotation angle. The femoral transcondylar axis, and the caudoproximal tibial line, a line between the most caudal points of the condylus medialis and lateralis tibiae, are used to calculate the femorotibial rotation angle.

The femoral varus (or valgus) angle:

The femoral varus or valgus angle was calculated as described by Dudley et al. (30) and Oxley et al. (40). Two axes needed to be defined: the femoral transcondylar axis, which is the line between the lateral and medial femoral condyle points, and the femoral longitudinal axis (Figure 2). Both vectors were projected into the dorsal femur plane where they form the varus or valgus angle of the femur.

**Figure 3:**
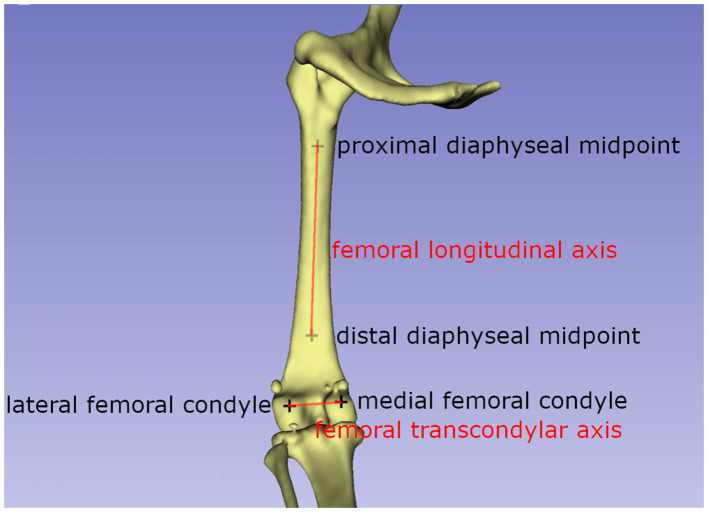
Schematic drawing explaining the axis used to calculate the femoral varus/valgus angle. The femoral transcondylar axis, a line between the medial and lateral femoral condyle, and the femoral longitudinal axis form the femoral valgus or varus angle.

The femorotibial rotation angle:

The method of Löer (19) was used to measure the femorotibial rotation (19, 23). According to this method, two vectors need to be calculated: the femoral transcondylar axis, as defined for the femoral antetorsion angle, and the caudoproximal tibial line, a line between the most caudal points of the condylus medialis and lateralis tibiae (Figure 3). The transverse tibial plane was defined by its normal vector, which is the tibial longitudinal axis and both vectors were projected in this plane. The tibia longitudinal axis was defined by two points situated on the proximal and distal midpoint of the tibial diaphysis.

**Figure 4:**
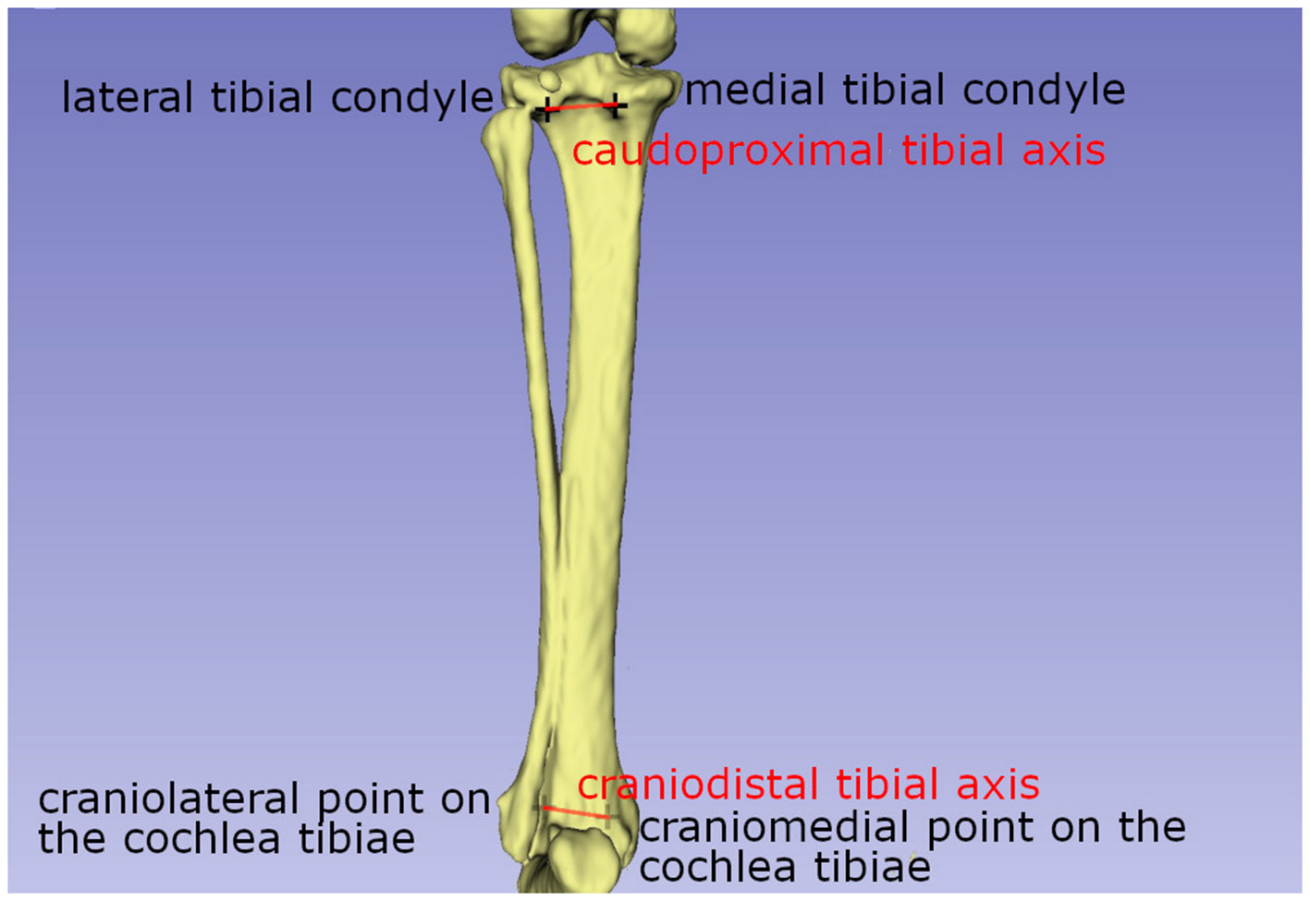
Schematic drawing explaining the axis used to calculate the tibial torsion angle. A line between the most cranial points on the lateral and medial part of the cochlea of the tibia, the distal tibial line, and the caudoproximal tibial line, are used to calculate the tibial torsion angle.

The tibial torsion angle:

Previous descriptions of tibial torsion angles by Löer (19) and Apelt (21) utilized the distal cranial tibial and proximal caudal tibial lines (19, 21, 23). The line between the most cranial points on the lateral and medial part of the cochlea of the tibia (Figure 4) was the distal tibial line. The caudoproximal tibial line was defined as the connection between the most caudal protuberance of the medial and the lateral tibial condyle (Figure 4). Both vectors were projected on the transverse tibial plane as defined above.

**Figure 5:**
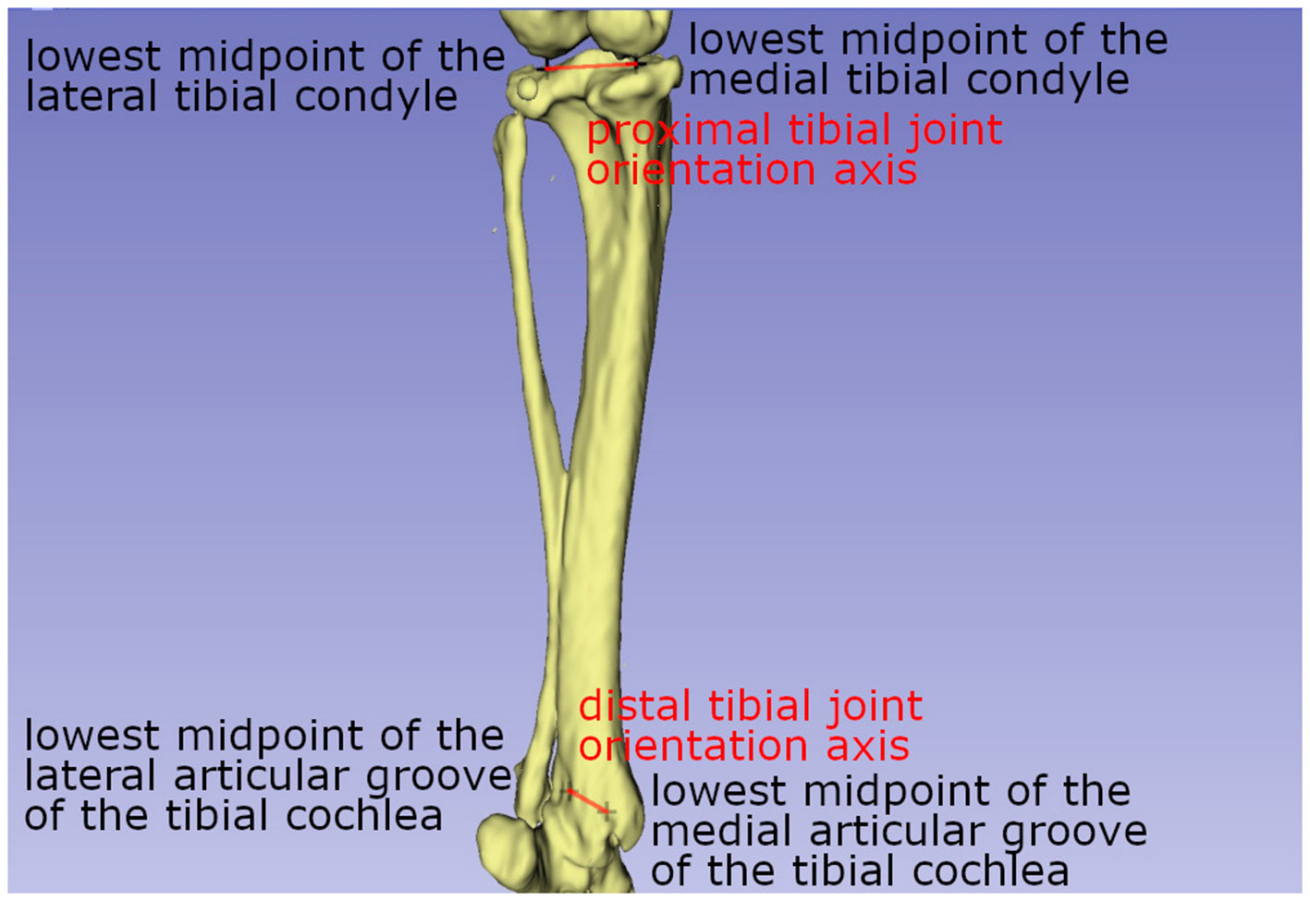
Schematic drawing explaining the axis used to calculate the tibial varus (or valgus) angle. The tibial varus (or valgus) angle was calculated with the help of a proximal tibial joint orientation line, a line between the lowest midpoint of the lateral and medial tibial condyle, and the distal tibial joint orientation line, a line between the lowest midpoint of the lateral and medial articular groove of the tibial cochlea.

The tibial varus (or valgus) angle:

A similar method to the method described by Newman et al. (11) was used to measure the varus or valgus angle of the tibia (11, 65). Proximal and distal tibial joint orientation lines were defined, proximally as the line between the lowest midpoint of the lateral and medial tibial condyle and distally as the line between the lowest midpoint of the lateral and medial articular groove of the tibial cochlea (Figure 5). Both vectors were projected on the dorsal tibial plane.

**Figure 6:**
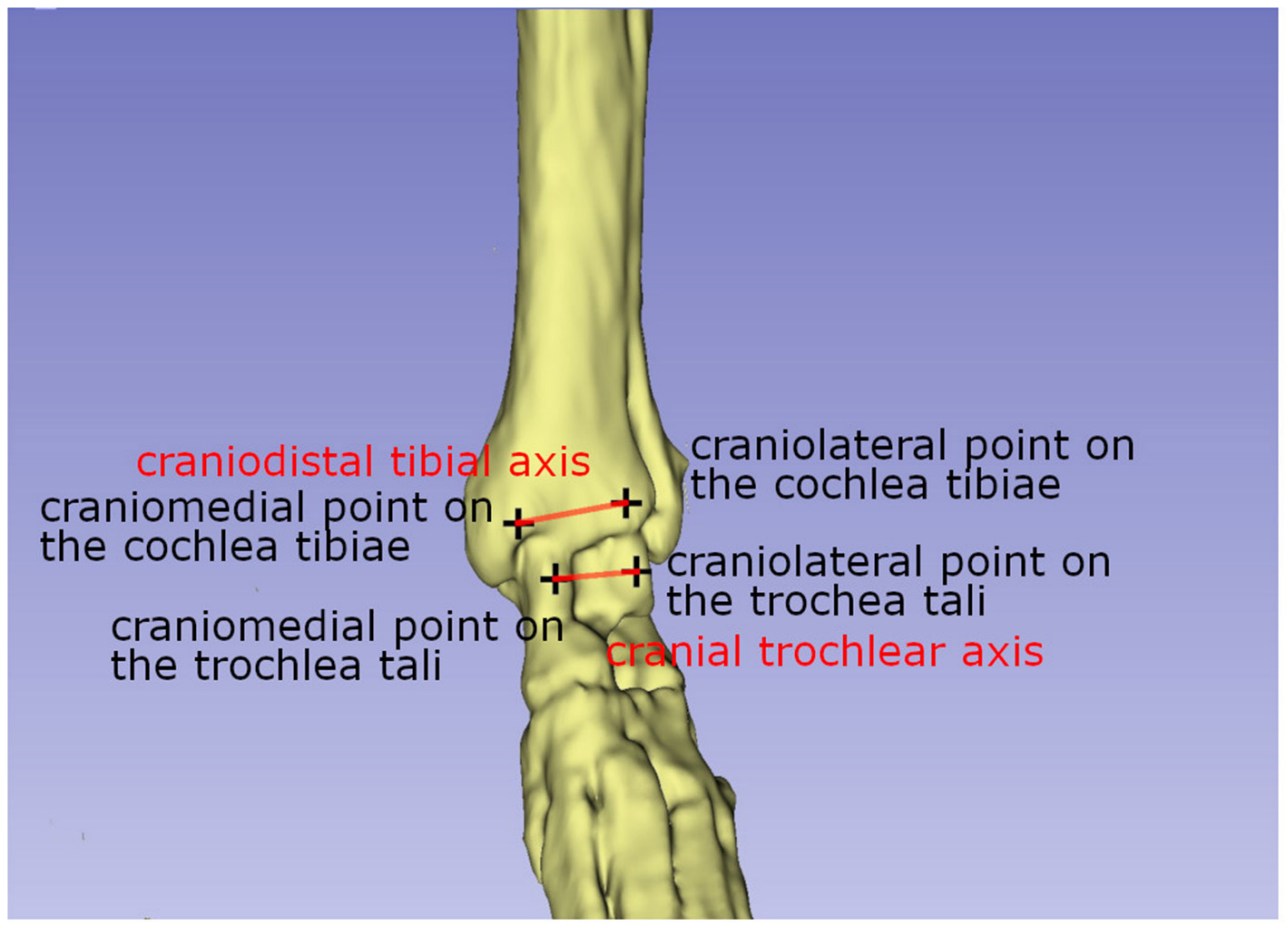
Schematic drawing explaining the axis used to calculate the tibiotalar rotation angle. The tibiotalar rotation angle was calculated as the angle between the craniodistal tibial line, and the cranial trochlear axis, a line connecting the medial and lateral most cranial point on the trochlea tali.

The tibiotalar rotation angle:

The tibiotalar rotation angle was calculated as the angle between the craniodistal tibial line, a line between the most cranial points on the lateral and medial part of the cochlea of the tibia, and a line connecting the medial and lateral most cranial point on the trochlea tali (Figure 6) (55). Both lines were projected on the transversal tibial plane.

Statistical analysis:

Boxplots were created using the MedCalc^®^ Statistical Software version 20.026 (MedCalc Software Ltd, Ostend, Belgium; https://www.medcalc.org; 2022). Each breed was compared to all others with SPSS (IBM Corp. Released 2020. IBM SPSS Statistics for Windows, Version 27.0. Armonk, NY: IBM Corp), using a general linear model with a Bonferonni adjustment.

## Results

**Figure 7:**
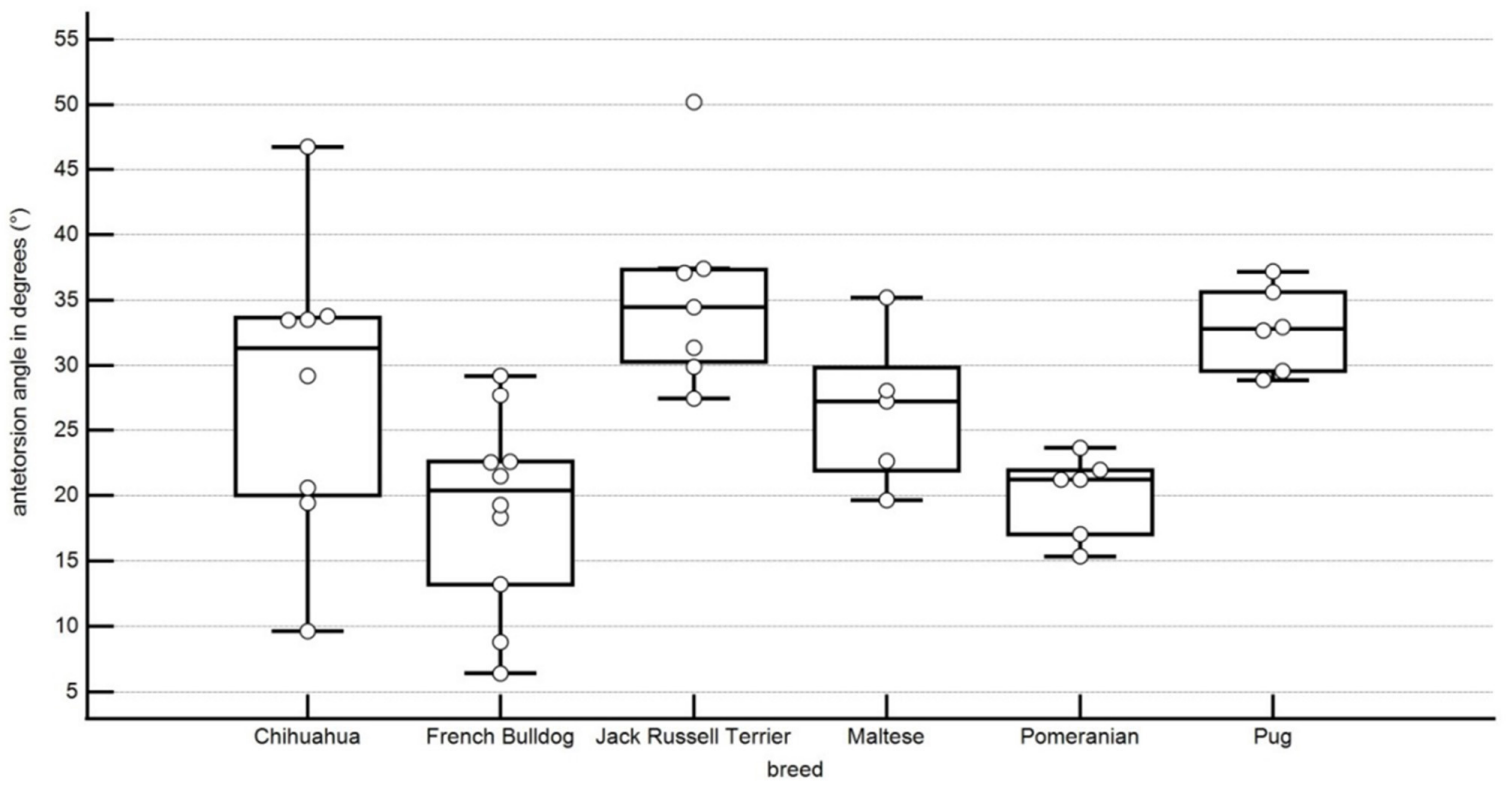
Boxplots representing the antetorsion angle in degrees in relation to the different breeds. Each point represents an angle of one pelvic limb. The bottom and top lines of the box represent the first and third quartiles, the band inside the box represents the median and the points outside the plots represent outliers.

In this study, the antetorsion angle was of 28.3° ± 10.7° in Chihuahuas, 20.1° ± 2.9° in Pomeranians, 35.4° ± 6.9° in Jack Russel Terriers, 32.8° ± 3.0° in Pugs, 19.0° ± 7.1° in French bulldogs, 26.6° ± 5.3° in Malteses (Figure 7). The difference was significant between the French bulldog and the Jack Russell Terrier, between the Jack Russell Terrier and the Pomeranian, between the Pug and the French bulldog, and between the Pomeranian and the Pug (Table 1).

**Table 1:** Pairwise comparison of the different breeds using a general linear model with a Bonferroni adjustment.

**Breed (I)**	**Breed (J)**	**Mean difference (I-J)**	**Standard error**	**df**	**Bonferroni-Sig**.	**95% Wald confidence interval of the difference**	
						**Lower**	**Upper**
Chihuahua	French Bulldog	9.35	4.45	1	0.29	−2.83	21.52
	Jack Russel Terrier	−7.13	4.68	1	0.64	−19.17	4.91
	Maltese	1.74	4.64	1	1.00	−8.03	11.51
	Pug	−4.52	3.89	1	0.98	−14.25	5.20
	Pomeranian	8.23	3.76	1	0.26	−2.23	18.69
French Bulldog	Chihuahua	−9.35	4.45	1	0.29	−21.52	2.83
	Jack Russel Terrier	−16.48	3.78	1	< 0.01	−27.29	−5.66
	Maltese	−7.60	3.74	1	0.29	−17.63	2.42
	Pug	−13.87	2.75	1	< 0.01	−21.82	−5.92
	Pomeranian	−1.12	2.55	1	1.00	−6.55	4.32
Jack Russel Terrier	Chihuahua	7.13	4.68	1	0.64	−4.91	19.17
	French Bulldog	16.48	3.78	1	< 0.01	5.66	27.29
	Maltese	8.87	4.00	1	0.27	−2.36	20.10
	Pug	2.61	3.10	1	1.00	−4.81	10.02
	Pomeranian	15.36	2.92	1	< 0.01	6.85	23.87
Maltese	Chihuahua	−1.74	4.64	1	1.00	−11.5	8.03
	French Bulldog	7.60	3.74	1	0.29	−2.42	17.63
	Jack Russel Terrier	−8.87	4.00	1	0.27	−20.10	2.36
	Pug	−6.27	3.05	1	0.29	−14.50	1.97
	Pomeranian	6.49	2.87	1	0.26	−1.66	14.64
Pug	Chihuahua	4.52	3.89	1	0.98	−5.20	14.25
	French Bulldog	13.87	2.75	1	< 0.01	5.92	21.82
	Jack Russel Terrier	−2.61	3.10	1	1.00	−10.02	4.81
	Maltese	6.27	3.05	1	0.29	−1.97	14.50
	Pomeranian	12.75	1.36	1	< 0.01	8.77	16.74
Pomeranian	Chihuahua	−8.23	3.76	1	0.27	−18.69	2.23
	French Bulldog	1.12	2.55	1	1.00	−4.32	6.55
	Jack Russel Terrier	−15.36	2.92	1	< 0.01	−23.87	−6.85
	Maltese	−6.49	2.87	1	0.26	−14.64	1.66
	Pug	−12.75	1.36	1	< 0.01	−16.74	−8.77

A difference between two breeds was considered as being significant when the p-value was under 0.05 [Bonferroni-significance (Bonferonni-Sig.) column].

**Figure 8:**
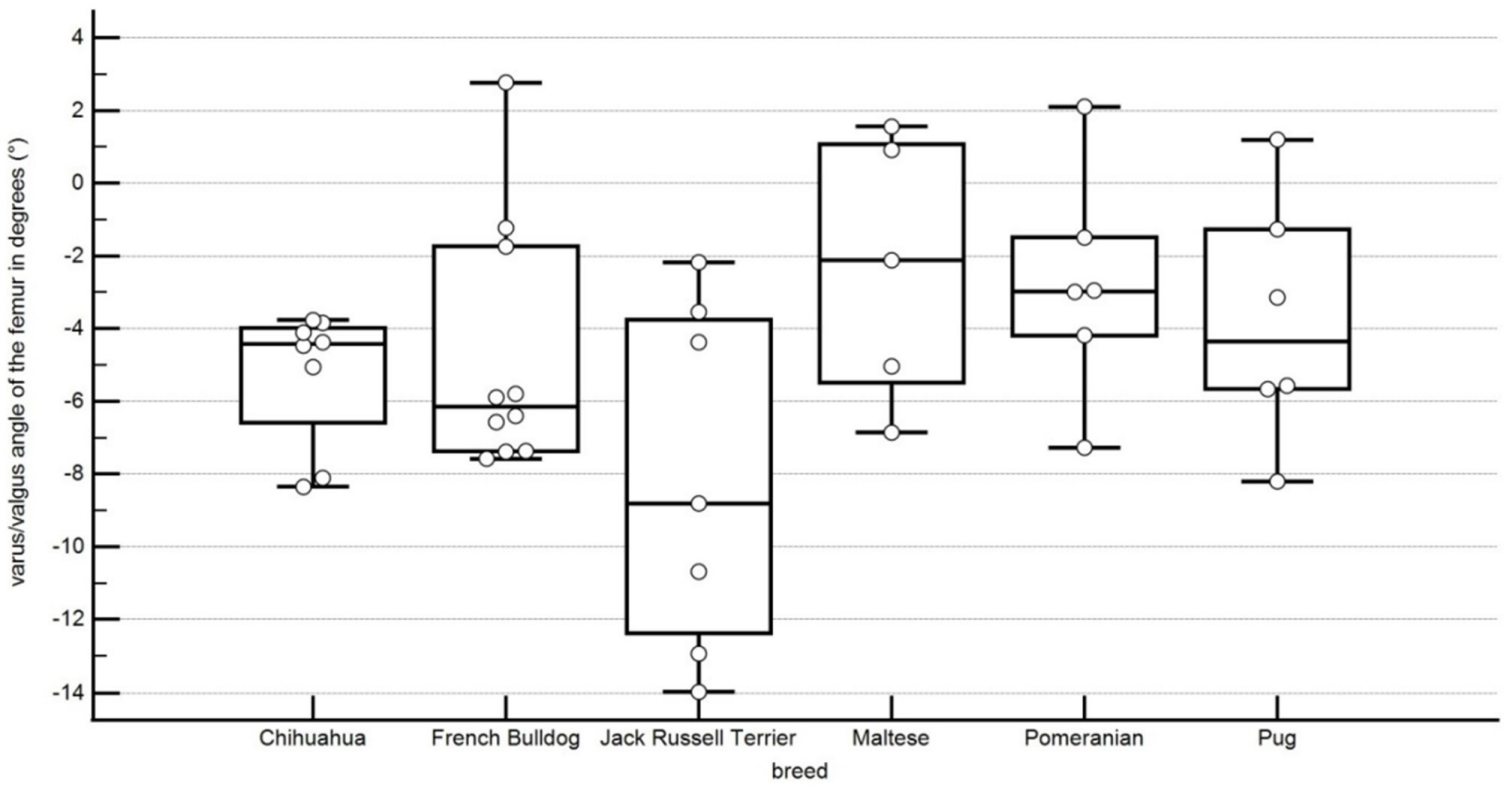
Boxplots representing the varus or valgus angle of the femur in degrees in relation to the different breeds. A negative angle represents a varus, and a positive angle represents a valgus. Each point stands for the angle of one pelvic limb. The bottom and top lines of the box represent the first and third quartiles, the band inside the box represents the median and the points outside the plots represent outliers.

In our population, the varus of the femur was of 5.3° ± 1.8° in Chihuahuas, 2.8° ± 2.8° in Pomeranians, 8° ± 4.4° in Jack Russell Terriers, 3.8 ° ± 3.1° in Pugs, 4.7° ± 3.3° in French bulldogs and 2.3° ± 3.3° in Maltese (Figure 8). These results suggest that there is considerable variation between breeds in the varus angle of dogs without medial patellar luxation. This difference was statistically significant between Chihuahuas and Malteses and Pomeranians, between Malteses and Pugs, between Pugs and Jack Russell Terriers, and between Pomeranians and Jack Russell Terriers (Table 1).

**Figure 9:**
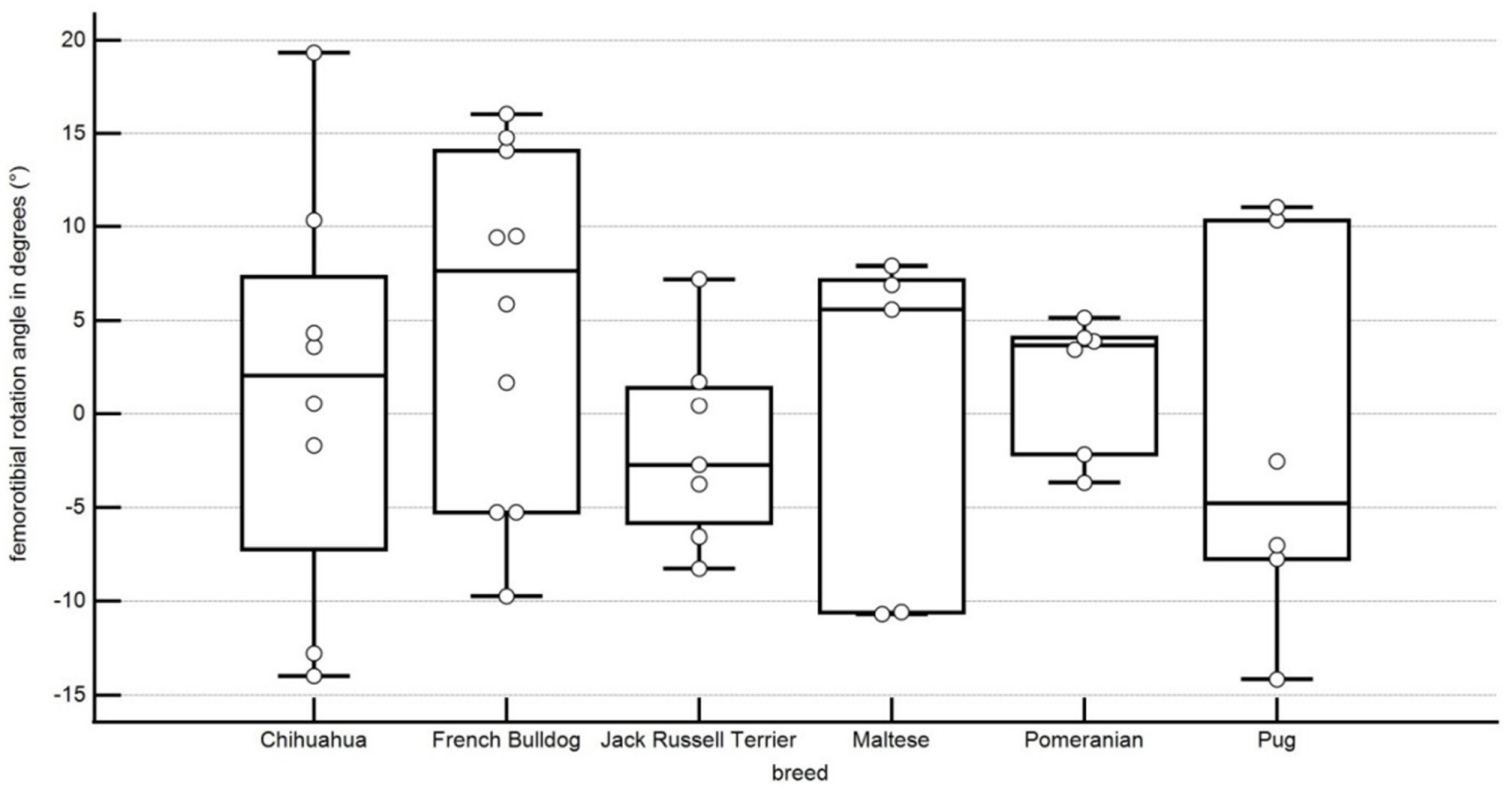
Boxplots representing the femorototibial rotation angle in degrees in relation to the different breeds. A negative angle represents an external rotation, and a positive angle represents an internal rotation. Each point represents the angle of one pelvic limb. The bottom and top lines of the box represent the first and third quartiles, the band inside the box represents the median and the points outside the plots represent outliers.

In this study, the tibial torsion was 5.5° ± 6.2° external torsion in Chihuahuas, 1.1° ± 4.1° internal torsion in the Pomeranians, 5.2° ± 9.5° external torsion in the Jack Russell Terrier, 6.1° ± 8.0° internal torsion in Pugs, 0.1° ± 5.9° external torsion in French Bulldogs, 9.2° ± 4.7° external torsion in Maltese (Figure 9). A statistical significance of the difference of values between the Chihuahua and the Pug, the Pomeranian and the Chihuahua, the French Bulldog and the Maltese, the Maltese and the Pug, and the Pomeranian and the Maltese was shown (Table 1).

**Figure 10:**
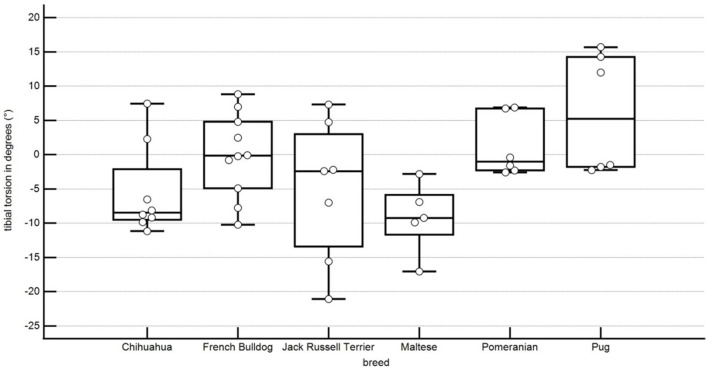
Boxplots representing the tibial torsion angle in degrees in relation to the different breeds. A negative angle represents an external torsion, and a positive angle represents an internal torsion. Each point represents the angle of one pelvic limb. The bottom and top lines of the box represent the first and third quartiles, the band inside the box represents the median and the points outside the plots represent outliers.

The tibial varus or valgus angle is here of 2.0° ± 2.9° valgus in Chihuahuas, 2.1° ± 2.7° valgus in Pomeranians, 6.4° ± 6.8° valgus in Jack Russell Terriers, 0.0° ± 5.7° valgus or varus angle in Pugs, 3.0 ° ± 5.8° valgus in French Bulldogs, 8.8° ± 8.6° valgus in Maltese. No statistical significance between the breeds could be shown (Figure 10; Table 1).

**Figure 11:**
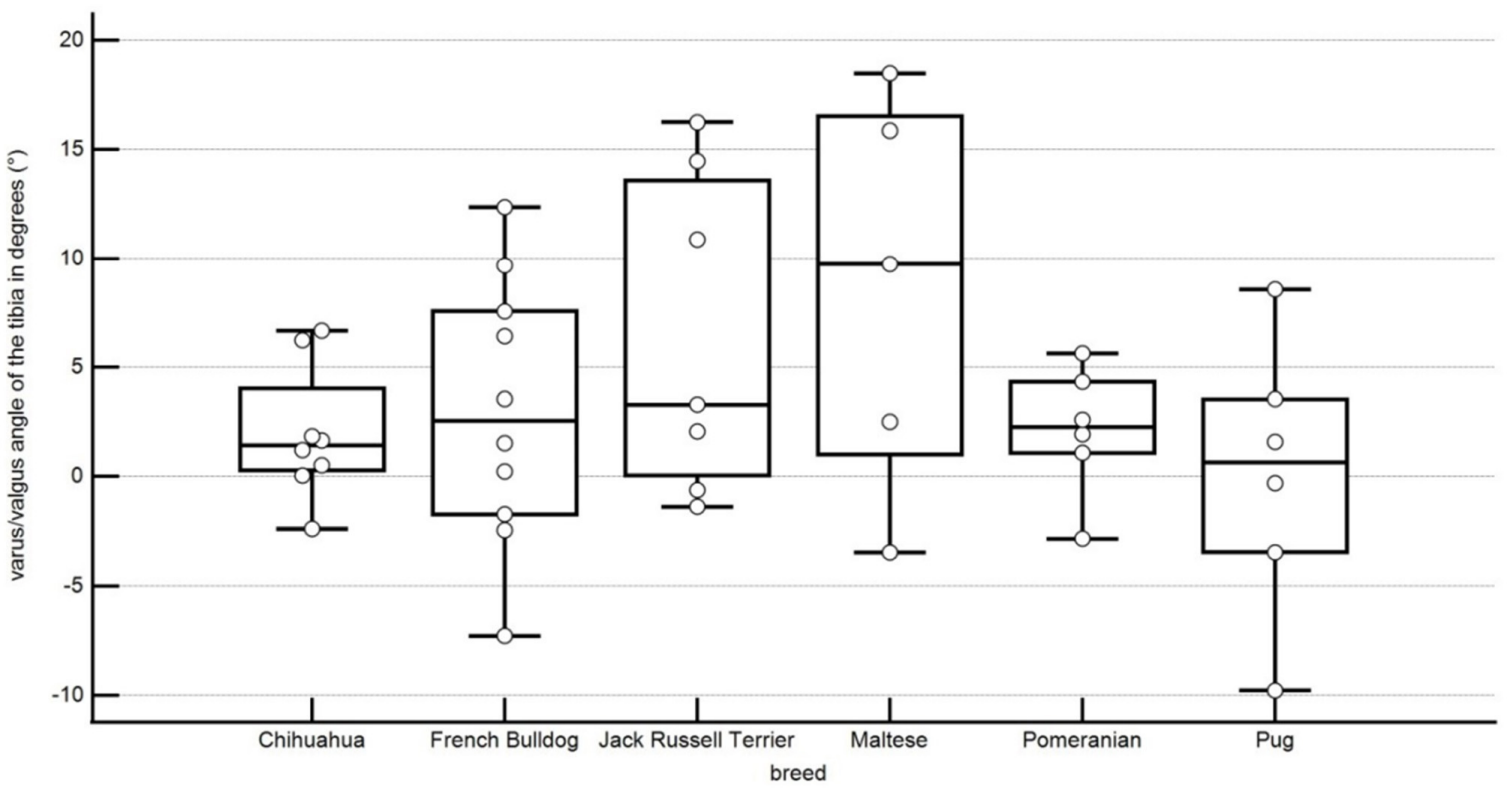
Boxplots representing the varus or valgus angle of the tibia in degrees in relation to the different breeds. A negative angle represents a varus, and a positive angle represents a valgus. Each point represents the angle of one pelvic limb. The bottom and top lines of the box represent the first and third quartiles, the band inside the box represents the median and the points outside the plots represent outliers.

In our population, the femorotibial rotation angle was 1.2° ± 10.4° rotation in Chihuahuas, 1.8° ± 3.4° rotation in Pomeranians, −1.7° ± 4.9° rotation in Jack Russell Terriers, −1.7° ± 9.4° rotation in Pugs, 5.1° ± 8.8° rotation in French Bulldogs, −0.2° ± 8.6° rotation in Maltese. No statistical significance between the breeds could be shown (Figure 11; Table 1).

**Figure 12:**
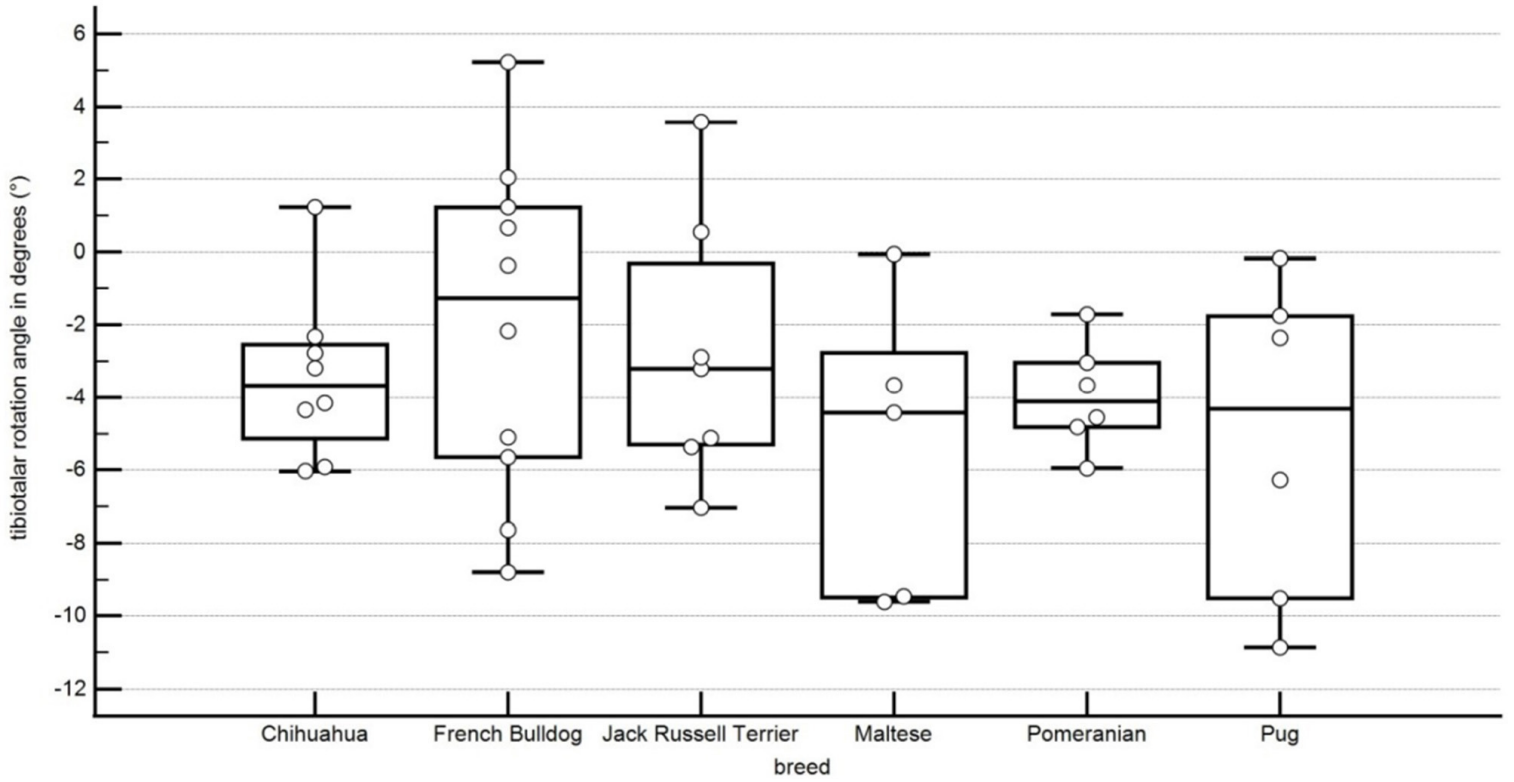
Boxplots representing the tibiotalar rotation angle in degrees in relation to the different breeds. A negative angle represents an external rotation, and a positive angle represents an internal rotation. Each point represents the angle of one pelvic limb. The bottom and top lines of the box represent the first and third quartiles, the band inside the box represents the median and the points outside the plots represent outliers.

In this study, the tibiotalar rotation angle was −3.4° ± 2.2° rotation in Chihuahuas, 1.1° ± 4.1° rotation in Pomeranians, −2.8° ± 3.4° rotation in Jack Russell Terriers, −5.2° ± 4.0° rotation in Pugs, −2.1° ± 4.4° rotation in French Bulldogs, −5.4° ± 3.7° rotation in Maltese. No statistically significant difference could be shown between the breeds (Figure 12; Table 1).

## Discussion

This study showed that there were significant differences in normal values for antetorsion, femoral varus or valgus, and tibial torsion across breeds (Table 1). There were no significant differences in the values of tibial varus or valgus, tibiotalar rotation, and femorotibial rotation between the different breeds (Table 1). Therefore, our hypothesis that canine pelvic limb alignment is breed-specific is partially correct.

The six investigated breeds were the French Bulldog, the Pug, the Pomeranian, the Maltese, the Jack Russell Terrier, and the Chihuahua. These breeds include chondrodystrophic, toy, and brachycephalic breeds, and all are prone to medial patellar luxation.

The femoral antetorsion angle showed high variation in previous studies using different canine breeds suggesting that femoral antetorsion angles could be breed-specific. Those studies all used CT-images and a transversal projection plane to measure the antetorsion angle, but differences due to other factors, like patient positioning, differences in measurement techniques, and in the measurement tools used could also play a role in the high angle variation. Reference values have been reported to be 26.1° ± 6.4° in Labrador Retrievers, 30.79° ± 4.24° in English Staffordshire Bullterriers and 19.8° ± 4.6° in Toy Poodles (11, 18, 66). The antetorsion angle does not depend on the length of the femur, which does not exclude the possibility of a breed-specific value because these studies included many different breeds (48, 54).

Femoral varus angles in dogs without orthopedic disease showed high differences in various CT studies using MPR to determine the dorsal plane (18, 30).

The femoral varus angle was reported to be 0.3° ± 2.8° in Toy Poodles and 8.8° ± 3.3° in large breeds (18, 30). The first study defined the varus or valgus angle as the angle between a perpendicular to a transcondylar axis and the longitudinal axis, and the second study defined the angle as the summation of two angles, the transcondylar angle, between a transcondylar axis and a horizontal line, and the proximal femoral axis angle, an angle between the femoral longitudinal axis and an orthogonal line (18, 30).

Pomeranian was the only breed in this project that had already been investigated in another study using dogs without orthopedic disease. The femoral varus angles in this study ranged from 2° to 13° (15), showing a clear difference to the 2° valgus to 7° varus measured in our study. The different results of both studies could be explained by the population of dogs, but also by differences in the measurement methods used. The angles were measured in both studies using an orthogonal axis to the transcondylar axis of the femur, and the femoral longitudinal axis, but we used CT-images, and they used radiographs. Many authors described the radiographic measurement of the femoral varus or valgus angle as not precise, because it depends on several factors of correct femoral positioning and should not be performed without fluoroscopy or other supporting technique to validate perfect femoral positioning prior to radiography (50–53, 57). For corrective femoral osteotomy, cut-off values of 10° or 12° femoral varus angle have been described (16, 57, 67). In our study, one of the Jack Russell Terriers without patellar luxation showed a varus angle of more than 13°. One possibility is that some breeds show a higher variation in the femoral varus or valgus angle than other breeds and therefore cut-off values might also be breed-specific. Additionally, increased femoral varus angles might be compensated by increased tibial valgus angles. Therefore, a comprehensive analysis of the whole hind limb might be necessary to conclude that a deformation could necessitate a corrective osteotomy. In this case, the patient had a higher tibial valgus angle of 16° on one side, but the same patient had a 1° tibial varus angle on the other side. These results should be investigated further to see if compensation explains these higher values. Perhaps these differences might also partially be the result of a variation in the setting of reference points, which could be tested using inter-observer and intra-observer agreement analysis, but the reference points were validated already in a prior study (27).

Bone torsion angles may correlate with, cause, or compensate for joint rotation angles, but further studies are required to determine their clinical relevance and physiological changes. The tibial torsion angle was reported to be of 9.1° ± 4.5° in Yorkshire Terriers, 7.24° ± 5.7° in English Staffordshire Terriers and 11.3° ± 4.3° external torsion in Toy Poodles (11, 18, 68). These authors used computed tomography, multiplanar reconstructed transversal planes and determination of tibial torsion using an angle between the proximal (caudal) and distal (cranial) tibial axes. The difference between the breeds was not as clear as in our study.

It is emphasized that the different angular values may be breed related as well as measurement related (11).

In this study, we used the same measurements for all breeds, thus demonstrating that differences do exist between breeds. Further research is required to compare canine breeds that are predisposed for patellar luxation and canine breeds that are not predisposed for patellar luxation as well as to establish normal reference values for various canine breeds.

One of the limitations of our study was the limited number of hindlimbs without medial patellar luxation in predisposed breeds. The low number of dogs was considered by the statistical analysis, but bias cannot be completely excluded. Dogs in this study came from private pet owners in Germany and France which presumably makes the results more valid than if they had come from a specially selected population. Since the gene pool of dog breeds might slightly vary in more or less separate populations, such as in different countries or continents, any reference values must be evaluated with caution from this point of view. Therefore, our obtained angular values should not be applied to surgical purpose, without further investigations on larger dog groups. Only small breeds predisposed to patellar luxation were included, so it remains possible that larger breeds or breeds not predisposed to patellar luxation may not show breed-specific values. In this project, we included femoral torsion, femoral varus (or valgus), femorotibial rotation, tibial torsion, tibial varus (or valgus) and tibiotalar rotation angles, based on their clinical relevance in veterinary orthopedic surgery. Bone deformities, including changes in distal femoral varus and torsion and tibial varus or valgus and torsion angles, are frequently described in the veterinary orthopedic literature, particularly in relation to medial patellar luxation (3, 45). Most breeds in our study showed significant differences in femoral anteversion and varus or valgus and tibial torsion angles. These angles are important for planning corrective femoral and tibial osteotomies (48, 58, 69), a procedure used to improve the prognosis of patients with a high degree of patellar luxation associated with bone deformities where routine surgical procedures such as tibial tuberosity transposition, femoral groove trochleoplasty and soft tissue rebalancing may not be sufficient.

No statistically significant difference could be found for the varus or valgus angle of the tibia, the femorotibial rotation angle, and the tibiotalar rotation angle. These angles also showed a high variance. Abnormal femorotibial rotation is part of the pathophysiology of patellar luxation (55). Therefore, joint rotation angles were included into this study for comprehensive evaluation of the whole hind limb alignment, but their clinical relevance, precision and accuracy are not determined yet and further research is required. Canine stifle joints should be rotationally stable in the extended position, but they allow slight rotation in the flexed position. In our study, hind limb positioning was not standardized based on the retrospective use of CT-data, and joint rotation angles might be influenced by the position of the hind limbs.

In human medicine femoral and tibial reference values are considered specific to the technique used and cannot simply be compared or transferred between different imaging modalities and measurement techniques (58). This probably also applies to veterinary medicine, where standardized positioning with fully extended and parallel hind limbs is even more difficult to achieve than in human medicine. Therefore, our results and angular values may not be transferable to other modalities and techniques. For this reason, we have developed a free downloadable plug-in for 3D Slicer that other veterinarians can use to perform three-dimensional angular measurements in canine hind limbs. Furthermore, as open-source software it can be modified and adapted to future changes in software and computer technology as well as to the latest research advances in veterinary orthopedics. We believe that in the future two-dimensional radiographic angular measurements might be replaced by three-dimensional techniques using computed tomography and this project might contribute to this development.

## Conclusion

In conclusion, we have shown that the antetorsion, tibial torsion and femoral varus or valgus angles of the canine pelvic limb alignment are breed-specific, and further studies may find additional significant differences between other breeds and in larger populations. The results of this comparison show the need to determine reference values for individual breeds. Reference values are especially important for patients which are bilaterally affected, and where one limb cannot be used as the reference for comparison.

## Data availability statement

The data analyzed in this study is subject to the following licenses/restrictions: Data contain animal and owner data. Requests to access these datasets should be directed to bruehschwein@lmu.de.

## Ethics statement

Ethical review and approval was not required for the animal study because the clinical data and CT-scans of the dogs were retrospectively selected and retrieved from hospital image archives and hospital information systems. All clinical data were collected, and all CT-scans were performed on dog patients during the clinical routine based on medical indications and with consent of the owner. Written informed consent for participation was not obtained from the owners because the study was retrospective. Therefore no written consents were obtained at the time of the study or analysis of the data. However, the owners were informed in advance. At the time of the dogs' examinations, all owners signed consent forms allowing a possible later scientific evaluation of the data and confirmed this with their signature. Retrospective analysis of the data was performed anonymously. Legal regulations of data protection regarding animal and owner data were always observed.

## Author contributions

JB-P: software programming, angular measurement data acquisition, analysis and interpretation of data, revision of the article for intellectual content, and final approval of the article. AM-L: idea of the study, revision of the article for intellectual content, and final approval of the article. MZ: CT-data acquisition, revision of the article for intellectual content, and final approval of the article. SR: advice, selection, calculation of statistical tests, revision of the article for intellectual content, and final approval of the article. AB: conception of the study, acquisition of CT-data, analysis and interpretation of data, drafting the manuscript, revision of the article for intellectual content, final approval of the article, and submission of the article. All authors contributed to the article and approved the submitted version.
